# Developing a device to determine the permeation of chemicals through whole protective boots

**DOI:** 10.1093/joccuh/uiaf031

**Published:** 2025-06-11

**Authors:** Hiroyuki Miyauchi, Shinobu Yamanoto, Takamasa Aoki

**Affiliations:** Department of Occupational Hygiene, School of Health Science, University of Occupational and Environmental Health, Kitakyushu, Japan; Department of Occupational Hygiene, School of Health Science, University of Occupational and Environmental Health, Kitakyushu, Japan; Organization for Health Support and Promotion of Environment and Safety, Kyushu Institute of Technology, Kitakyushu, Japan

**Keywords:** chemical-protective boots, permeation time, permeation test, toluene, whole chemical resistant

## Abstract

**Objectives:**

To develop a device to evaluate the permeation resistance of chemical-protective boots continuously in contact with liquid chemicals, and to compare the permeation time of the component material test pieces with the permeation time of the whole boot and thus evaluate its performance.

**Methods:**

The permeation time was calculated for toluene, dichloromethane, and acetone in 4 types of boot, according to Japanese Industrial Standard (JIS) T 8117:2005.

**Results:**

The permeation test for whole boots showed shorter permeation times than those of the component materials, according to JIS T 8117:2005. The permeation time of toluene was more than twice that of boots C and D. The permeation time of dichloromethane was more than twice that of boots A and C and more than 3 times shorter for boot A.

**Conclusions:**

The differences between the whole-boot tests and the material tests were thought to be related to variations in thickness, type of material, difference in adhesion, and penetration from pinwheels. This method enables the determination of the permeation of chemicals and other performance characteristics of the whole boot, which cannot be determined using testing of material specimens alone. By using this device for testing, appropriate boots that are protective against specific chemicals and can prevent chemically induced damage to the feet can be more efficiently selected.

## Teaser

Key points

Chemical-protective boots are important for protecting the feet when workers are handling chemicals. However, such boots have joints between the body and sole, and there are differences in the thicknesses of the boot parts. Therefore, the permeation resistance of chemical-protective boots as a whole may differ from the permeation resistance of the component materials when the boots are actually used. Consequently, we developed a device to evaluate the permeation resistance of all chemical-protective boots. The results of the permeation tests of the chemical-protective boots with toluene and dichloromethane showed that the permeation times were shorter for the whole boot than for component material test pieces. These results indicate that determining the permeation resistance can aid in selecting appropriate chemical-protective boots and prevent foot damage caused by chemical substances.

## 1. Introduction

In recent years, many accidents caused by corrosive chemicals that damage feet, lower limbs,^1^ and boots have been reported.^2-6^ The use of chemical-protective boots is important to prevent injury. However, it is difficult to determine whether information regarding the selection of appropriate chemical-protective boots is sufficiently communicated to users. For the materials used in chemical-protective boots, Japanese Industrial Standards (JIS) T8117:2005 presents a performance evaluation method using test pieces of component boot materials with a permeation test device.^7^ The breakthrough time is defined as the time when the permeating gas velocity reaches 0.1 μg/cm^2^/min. Permeation resistance is evaluated using the breakthrough time as the permeation time. In this method, the test pieces of boot materials used in the performance evaluation must be sampled from the thinnest parts of the sole, trunk, and upper parts of the boot. Alternatively, test pieces must be made of the same material with the same thickness as the thinnest part of the product. However, chemical substances may permeate not only through the material of the boot but also through adhesion and joints. Therefore, we developed a device that can evaluate the permeation performance of boots in continuous contact with liquid chemicals, and the permeation times of test pieces were compared with that of the whole boot to evaluate its performance.

## 2. Methods

### 2.1. Developing a permeation test device for whole chemical-protective boots in continuous contact with liquid chemicals

**Figure 1:**
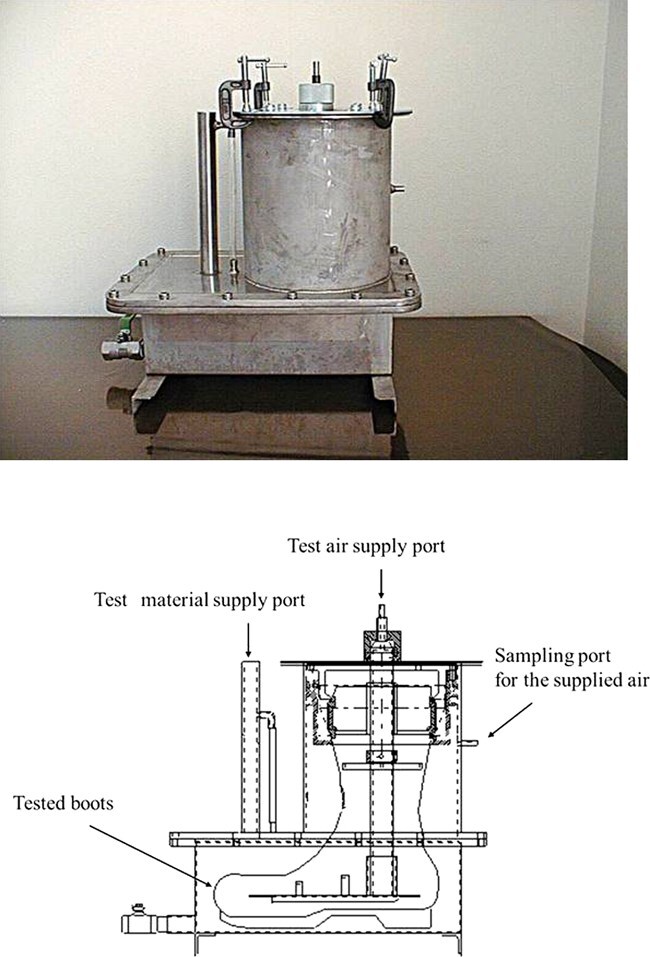
Appearance and structural drawing of the test device. The test substance is introduced via the supply port, fresh air is introduced through the test air supply port, and the air that exits from the sampling port is taken as a analytical sample.

Figure 1 shows the appearance and structural drawings of the test device. The purpose of this device is to measure the time required for the test chemical to permeate through the boots as a gas and the amount of permeated gas. As shown in Figure 1, space was provided up to the toe of the boots to reflect the permeation amount of the test chemicals. The permeation times of the test pieces were compared with the permeation time of the whole boots to evaluate their performance by placing the boots in the test device and then placing them in the thermostatic chamber.

**Figure 2:**
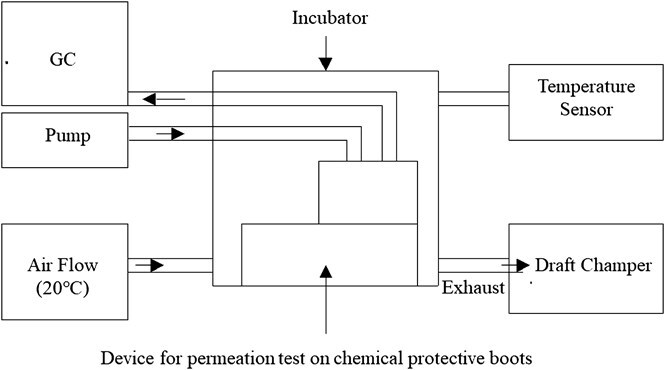
Overall schematic diagram of the test apparatus. Air at 20°C is supplied to a constant-temperature chamber installed inside a permeation test device, and then exhausted into a draft chamber.

The thermostatic chamber contains a temperature control unit (APISTE PAU UNIT). The temperature sensor installed in the temperature control unit was set to maintainthe internal temperature of the thermostatic chamber at 20°C, which was the test condition. As shown in Figure 2, the thermostatic chamber was designed to supply and exhaust air in a certain direction rather than circulating air, with the temperature adjusted by the temperature control unit, to prevent an increase in concentration due to the gas of the leaked test chemical. The internal temperature was monitored during the test using a temperature recorder (A&D THERMO RECORDER TR-72S).

Before starting the experiment, approximately 12 L of the test chemical substance was injected into the test device through the supply port to immerse the whole boot in a chemical of the device. Fresh air was supplied through a fresh-air supply port at a flow rate of 1 L/min. The air flowing out of the gas sampling port of the test device was evaluated over time. The concentrations of the test chemical in the sampled air were analyzed using gas chromatography (GC–MS, Agilent 6890 N). The experiment was conducted 3 times in accordance with JIS T8117:2005^7^ to determine the arithmetic mean value.

### 2.2. Protective boots for use against chemicals and tested chemical substances

The boots tested for protection against chemicals are listed in Table 1. Four types of boots were used against the chemicals tested: chemical-protective boots A and B were made of polyvinyl chloride (PVC), and boots C and D were made of natural rubber and synthetic rubber (Table 1). Toluene, dichloromethane, and acetone were used as the test substances. The test method was in accordance with JIS T8117:2005.^7^ The breakthrough time is defined in JIS T8117:2005 as the time it takes for the transmitted gas velocity to reach 0.1 μg/cm^2^, the breakthrough time is expressed in terms of permeation time and presents the permeation resistance.

**Table 1:** Characteristics of the tested boots used for protection against chemicals.

**Boots**	**Materials**		**Tested surface area, cm** ^**2**^	**Thickness, mm**		
	**Body**	**Soles**		**Body**	**Soles**	
					**Arch**	**Heel**
**A**	Polyvinyl	Polyvinyl	2549	1.9	15	15
**B**	Polyvinyl	Polyvinyl	2135	2.1	15	15
**C**	NR, SR	NR, SR	2102	2.1	11	21
**D**	NR, SR	NR, SR	1616	1.8	13	25

Abbreviations: NR, natural rubber; SR, synthetic rubber.

## 3. Results

**Figure 3:**
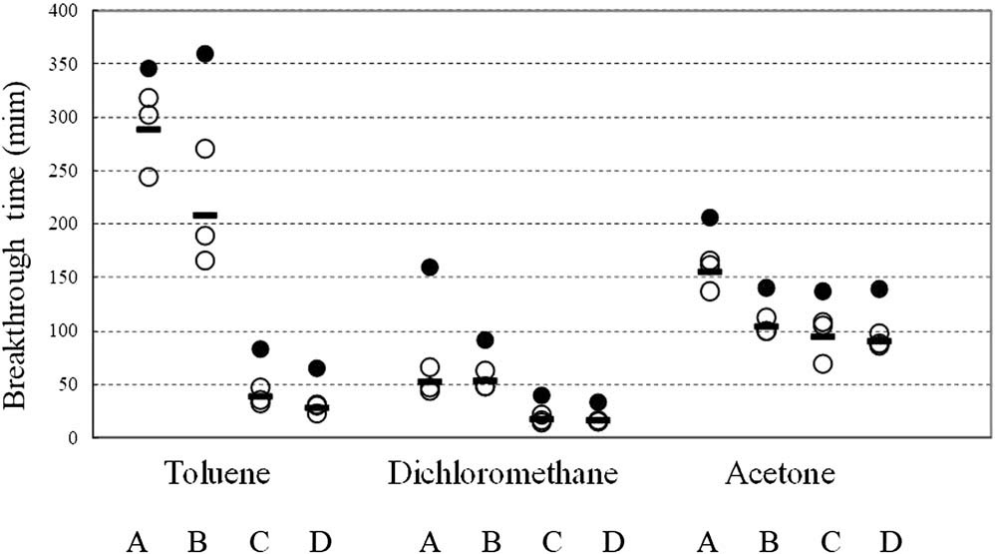
Breakthrough times of 3 types of substance on test boots A-D. Experimental values of transmission times for 3 different materials for 4 different types of boots are shown. The plotted values are of 3 repeated transmission tests of the whole protective boots and their arithmetic mean, and the permeation time of the component materials according to the method of Japanese Industrial Standard (JIS) T8117:2005. Legend: ● experimental value of material; — experimental value of whole (mean); ○experimental value of whole.

Table 2 displays the results of the experiment on the whole boot using this device, the results of the specimen component material test conducted according to JIS T8117:2005,^7^ and the data for the permeation test published by the manufacturer. Figure 3 shows the breakthrough times of the component materials and the whole boot for the test substances. The results for the whole boot showed that the permeation time was shorter than for the component materials according to JIS T8117:2005^7^ for toluene, dichloromethane, and acetone. The permeation times of toluene through the whole boot for boots C and D were more than twice that for the material according to JIS T8117:2005.^7^ Similarly, for dichloromethane, the permeation times for the whole boot for boots A and C were more than twice as long. Notably, the permeation time of dichloromethane for boot A was less than one-third that observed in the material test.

**Table 2:** Results of the whole-boot and specimen material experiment and published permeation times of tested boots.

**Boot**	**Permeation time, min**								
	**Toluene**			**Dichloromethane**			**Acetone**		
	**Experiment**	**Published data for material**	**Experiment**	**Published data for material**	**Experiment**	**Published data for material**			
	**Whole-boot, mean (*n* = 3)**	**Specimen material**	**Whole-boot, mean (*n* = 3)**	**Specimen material**	**Whole-boot, mean (*n* = 3)**	**Specimen material**			
**A**	288	345	180	52	159	171	154	180	180
**B**	208	359	281	53	91	72	104	140	136
**C**	38	77	60	17	34	15	94	137	137
**D**	28	64	30-60	15	28	10-30	90	139	120-240

**Figure 4:**
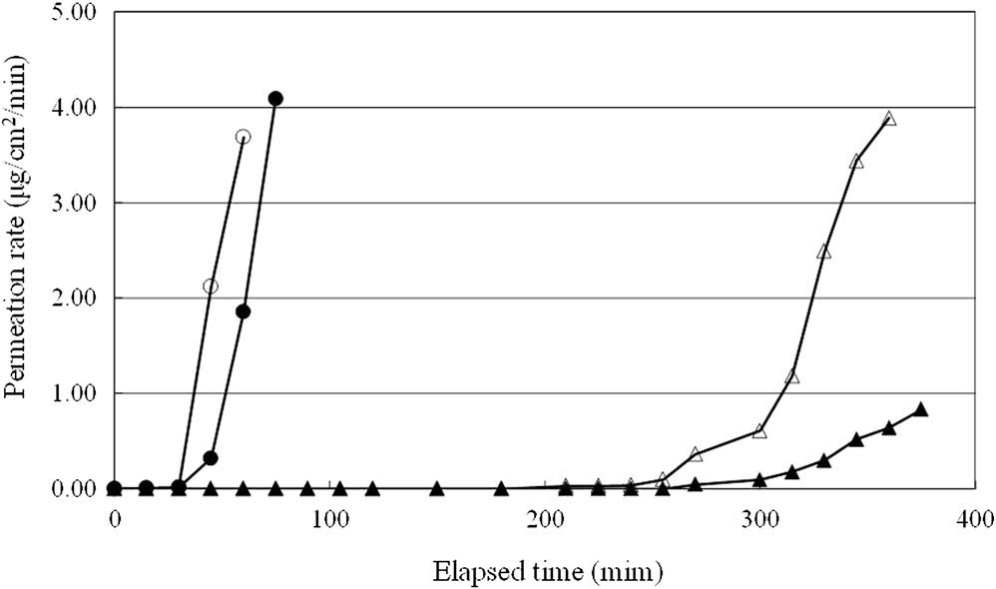
Breakthrough detection curves for the whole-boot permeation rates of one example on boots A-D for toluene. The breakthrough detection curves show the permeation rate of change with elapsed time during a whole-boot test of boots A-D (permeation time is taken at permeating rate reached 0.1 μg/cm^2^/min). Legend: ▲ = a, △ = B, ● = C, ○ = D.

Figure 4 shows the breakthrough detection curves for the permeation rates of the whole test of boots A-D for toluene. The slopes of the permeation rate over time for boots C and D began to increase sharply 30 minutes after initialization, whereas those for boots A and B began to increase more gradually after 255 and 225 minutes, respectively.

Visual observations of the boots after the experiment indicated that part of the surface of boot A swelled in response to toluene. The surfaces of boots A and B deteriorated in response to dichloromethane. Peeling of the sole and joint was observed in boot B. In boot C, the boot material dissolved in dichloromethane, and the color changed to that of the boot material.

## 4. Discussion

The results of this study showed that for boots C and D the permeation time of the whole boot for toluene was less than half that of a component material test piece according to JIS standards. In addition, the slopes of the permeation rates of boots C and D increased rapidly 30 minutes after the experiment began, whereas the slopes of boots A and B increased slowly from 255 and 225 minutes, respectively. Substantial variations were observed between the different boots.

JIS T8116:2005 (chemical-protective gloves) specifies the main performance tests as permeation test, penetration test, and deterioration test. In this study, a permeation test was performed to confirm that chemical substances that make contact with the surface of the material of chemical-protective boots are absorbed, diffuse inside the boots at the molecular level, and leave from the back of the boots. In general, the factors that affect the permeation of chemical substances through materials include the affinity between the chemical substance and the material, the thickness of the material, and the temperature. In addition, if the material itself has pinholes, the chemical substance may enter the inner surface and be detected due to penetration not permeation. In such cases, performance tests may be conducted using not only permeation tests but also penetration tests. Furthermore, if there are pinholes at joints or adhesion points between materials, or if pinholes occur owing to deterioration, chemicals may be detected on the inside, and degradation tests may be performed.

It is thought that the reason for the large difference in results between whole-contact testing of chemical-protective boots and the JIS standard material test for each boot is that whole-contact testing includes not only permeation through the material but also penetration through adhesives and joints and chemical penetration inside the boots due to degradation. The difference in the thicknesses of the soles of boots C and D was also considered a factor.

On the other hand, the main reason for the difference in results between boots C and D versus boots A and B in both the whole-boot contact test and the JIS standard material test was that boots A and B were made of PVC, whereas boots C and D were made of natural rubber and synthetic rubber, respectively, which are permeable to toluene. Furthermore, boots A and B are resistant to permeation by toluene, but not resistant to permeation by dichloromethane, albeit more resistant than boots C and D. These facts suggest the importance of selecting materials appropriate for the chemicals used.

Currently, JIS T8116:2005 (chemical-protective gloves) specifies a penetration test in addition to a permeability test to evaluate leakage at glove joints; a degradation test to determine the physical properties is also provided, if necessary. On the other hand, JIS T8117:1998^7^ for chemical-protective boots was revised to JIS T8117:2005^8^ with the addition of a permeability test in accordance with the recent addition to the American Society for Testing and Materials (ASTM) test method.^9^ However, JIS T8117:2005^9^ is only applicable to test specimens of component materials and does not consider permeation via the laminated part of the boot body or the joint between the body and sole.

Nevertheless, because of the complicated shape of chemical-protective boots, it is difficult to conduct penetration tests on the whole boot, including the joints. Therefore, JIS T8117:2005 (chemical protective boots) specifies only permeation tests for materials. Thus, JIS T8117:2005^9^ applies only to test specimens of materials and does not consider penetration between the body and soles.

The results for boots C and D were approximately half the permeation times of the material specimens indicated by the manufacturer, which suggests that the usable time could be incorrect if the value was based only on the performance evaluation experiment of the boot material. The usable time should account for permeation via lamination of the boot body and the joint between the body and sole.

To address these issues, in this study, we developed an evaluation method for the protective performance of whole boots in contact with chemical substances and compared the permeation performance of the whole chemical-protective boots for different chemical substances.

In a study on the permeation resistance of chemical-protective gloves, Miyauchi et al^10^ conducted permeation tests according to international standards (ISO) for 4 organic solvents on the main chemical-protective glove materials used in Japan and reported differences from the results of conventional degradation tests. Aoki et al^11^ conducted permeation tests on laminate films composed of new glove materials and described their usefulness. Furthermore, Aoki et al^12^ conducted an experiment on the permeation of a specimen of chemical protection glove material and on the entire glove, and the results differed, suggesting that the material alone was not sufficient for performance evaluation. They reported a discrepancy between the results of the permeation experiments on the test piece of the glove material and the entire glove. They estimated that one of the causes was the difference in thickness, which depended on the part of the glove.

Article 594 of the Industrial Safety and Health Regulations (Protective Equipment for Preventing Skin Disorders) was revised in April 2024. In Article 2, Paragraph 1, it became mandatory for workers who handle 1064 chemical substances (chemical substances causing skin disorders, etc) that are known to damage or sensitize the skin or eyes directly or to be absorbed or penetrate through the skin and affect the health of the whole body to wear appropriate personal protective equipment such as impermeable protective clothing, gloves, boots, and glasses.^13^

In February 2024, the Ministry of Health, Labor, and Welfare published a list of chemical substances that cause skin damage, and glove materials resistant to those chemical substances.^14^ Simultaneously, the Ministry of Health, Labor, and Welfare published version 1 of the Selection Manual for Protective Equipment to Prevent Skin Damage.^15^ The manual describes the selection of chemical-protective gloves.

The permeation test conducted in this study was subject to the limitations of conducting tests with just 4 types of boots and 3 different chemicals. In addition, the test was performed on only 1 lot. This is a limitation of this study, and it is important to confirm this finding in more detail. We hope that more detailed information will be provided on the selection of clothing and boots for chemical use in the future.

## 5. Conclusions

Our device was designed to reflect the performance of chemical-protective boots by means of a permeation experiment on the whole boot. Using this device for testing, it is possible to select chemical-protective boots that are appropriate for the substances used. Consequently, chemically induced damage to the feet can be prevented more efficiently.

## Data Availability

The data used and analyzed during this study are available from the corresponding author upon reasonable request.
